# Supplementary material for: Roles for DNA polymerase δ in initiating and terminating leading strand DNA replication
Source: Nat Commun. 2019 Sep 5;10:3992. doi: 10.1038/s41467-019-11995-z (PMC6728351; doi:10.1038/s41467-019-11995-z)
Supplement: Supplementary file 1 — Supplementary Information [file 41467_2019_11995_MOESM1_ESM.pdf]

Supplementary Information
for

Roles for DNA polymerase δ in initiating and terminating leading strand DNA replication

Zhou et al.

A

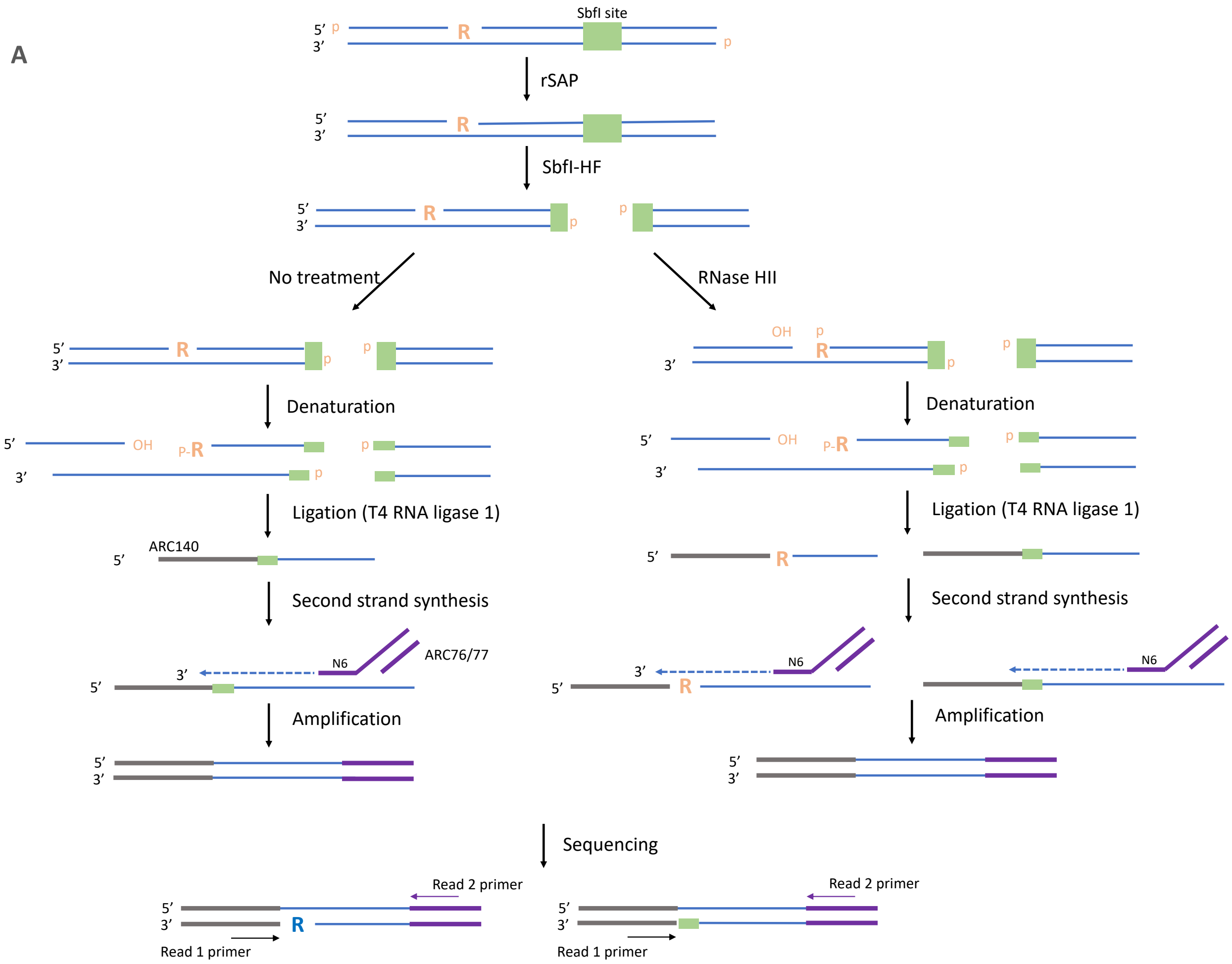

B

Modifications	Improvements
5’ blocking by rSAP	Reduce hydrolysis independent noise
No-treatment control	Control for hydrolysis-independent noise
E. coli RNase HII instead of KOH	Increased specificity towards single ribonucleotides[2]
SbfI restriction digestion	Provide internal standard for normalization between datasets

C

	RNase HII-HydEn-seq	PU-seq
Hydrolysis	RNase HII	KOH
5’ Capture	ssDNA ligation (T4 RNA ligase 1)	NEB NEXT Ultra hairpin adaptor
3’ Capture	Random hexamer	NEB NEXT Ultra hairpin adaptor
Preservation of strand polarity	Sequential end capture (5’ ligation followed by 3’ capture by hexamer)	Uracil incorporation on second strand and cleavage by USER enzyme, followed by sequencing indexing
Normalization	SbfI ends	Total reads

Supplementary Figure 1.

RHII-HydEn-seq library construction. **a**, RHII-HydEn-seq library preparation steps. Refer to Methods section for detailed description. Orange letter "R" denotes the location of ribonucleotide. Adaptor sequences ARC140 and ARC76/77 are indicated by grey and purple lines, respectively. The ribonucleotide is identified by the first base of read 1 by Illumina sequencing denoted by blue "R" (in contrast to the orange "R" because rNMPs in the template DNA were converted to dNMPs during PCR amplication), after exclusion of SbfI sites. **b**, Main modifications and their purposes in comparison to the original HydEn-seq (Alk-HydEn-seq) protocol[1]: 1) Because mechanical shearing during genomic DNA preparation could result in random ligatable 5' ends contributing to background noise, we added a phosphatase (rSAP) treatment to remove potential 5' phosphate before hydrolysis. 2) Inclusion of non-treatment samples allow us to further control for hydrolysis-independent noise. Signals from non-treatment controls are subtracted from RNase HII-treated samples during data analysis for noise removal. 3) Alkaline hydrolysis is not specific to ribonucleotide. Some other non-canonical nucleotides such as abasic sites are also prone to alkaline hydrolysis. *E. coli* RNase HII has been shown to preferentially cleave at signal ribonucleotides and thus serves as an ideal tool to specifically target single embedded ribonucleotides[2]. 4) We treated the genomic DNA with a restriction enzyme SbfI-HF which creates defined ends across the genome which can be captured by RHII-HydEn-seq. These SbfI-HF-dependent ends serve as internal standards that can be used to normalize between datasets and thus allow us to quantitatively compare different RHII-HydEn-seq datasets. **c**. Comparison between RNase HII-RHII-HydEn-seq and PU-seq. The current formats of the two ribonucleotide mapping techniques differ by choice of hydrolysis, end captures, preservation of strand polarity information and availability of internal standards. A

detailed comparison of all published ribonucleotide mapping technologies was described by Jinks-Robertson and Klein[3].

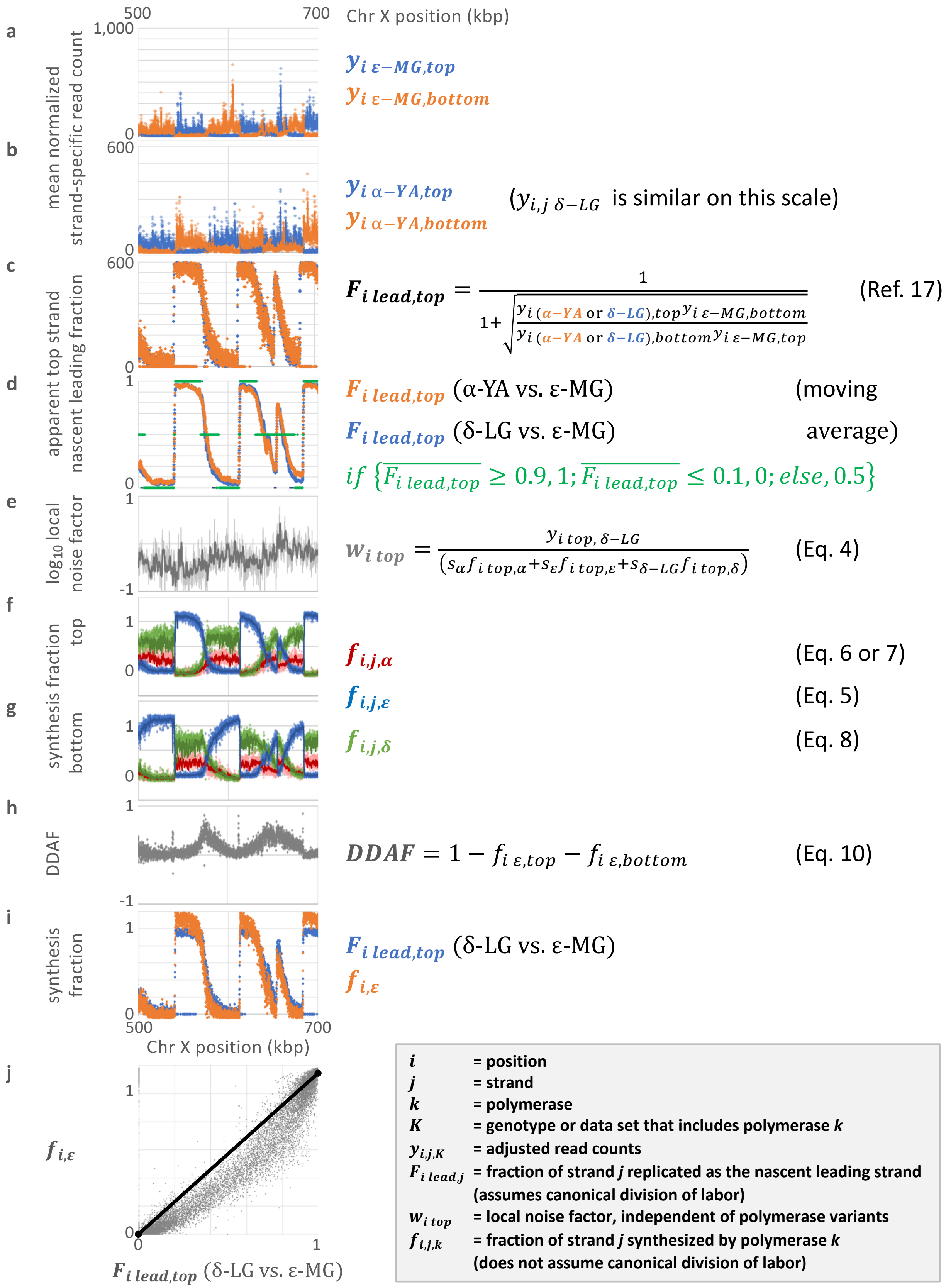
a
b
c
d
e
f
g
h
i
j
500
700
Chr X position (kbp)
1,000
0
600
mean normalized strand-specific read count
$y_{i\ \varepsilon-MG,top}$
$y_{i\ \varepsilon-MG,bottom}$
$y_{i\ \alpha-YA,top}$
$y_{i\ \alpha-YA,bottom}$
($y_{i,j\ \delta-LG}$ is similar on this scale)
apparent top strand nascent leading fraction
$F_{i\ lead,top} = \frac{1}{1+\sqrt{\frac{y_{i\ (\alpha-YA\ \text{or}\ \delta-LG),top}\, y_{i\ \varepsilon-MG,bottom}}{y_{i\ (\alpha-YA\ \text{or}\ \delta-LG),bottom}\, y_{i\ \varepsilon-MG,top}}}}$
(Ref. 17)
$F_{i\ lead,top}$ (α-YA vs. ε-MG)
$F_{i\ lead,top}$ (δ-LG vs. ε-MG)
(moving average)
$if\ \{\overline{F_{i\ lead,top}} \geq 0.9, 1; \overline{F_{i\ lead,top}} \leq 0.1, 0; else, 0.5\}$
$\log_{10}$ local noise factor
-1
$w_{i\ top} = \frac{y_{i\ top,\ \delta-LG}}{(s_\alpha f_{i\ top,\alpha} + s_\varepsilon f_{i\ top,\varepsilon} + s_{\delta-LG} f_{i\ top,\delta})}$
(Eq. 4)
synthesis fraction
top
bottom
$f_{i,j,\alpha}$
(Eq. 6 or 7)
$f_{i,j,\varepsilon}$
(Eq. 5)
$f_{i,j,\delta}$
(Eq. 8)
DDAF
$DDAF = 1 - f_{i\ \varepsilon,top} - f_{i\ \varepsilon,bottom}$
(Eq. 10)
synthesis fraction
$F_{i\ lead,top}$ (δ-LG vs. ε-MG)
$f_{i,\varepsilon}$
$f_{i,\varepsilon}$
$F_{i\ lead,top}$ (δ-LG vs. ε-MG)
i = position
j = strand
k = polymerase
K = genotype or data set that includes polymerase k
$y_{i,j,K}$ = adjusted read counts
$F_{i\ lead,j}$ = fraction of strand j replicated as the nascent leading strand (assumes canonical division of labor)
$w_{i\ top}$ = local noise factor, independent of polymerase variants
$f_{i,j,k}$ = fraction of strand j synthesized by polymerase k (does not assume canonical division of labor)

Supplementary Figure 2.

Illustrated calculation steps. See Methods for all equations. (**a**-**i**) Outputs of various calculations using RHII-HydEn-seq end densities on *S. cerevisiae* chromosome X from 500 to 700 kbp. Variables in the right column refer to equations found in Methods or in reference 35. (**a**-**b**) Read counts, per strand, averaged over all data sets with the same genotype (**a**, *pol2-M644G rnh201*Δ; **b**, *pol1-Y869A rnh201*Δ), untreated background samples subtracted from treated samples ($y_{i,j,K}$ in Eq. 3). The underlying data sets were first normalized against internal controls. (**c**-**d**) Estimates of the fraction of the cellular population in which the top strand is replicated as the nascent leading strand (**c**, unsmoothed; **d**, moving average). The calculations [4] are only accurate where the canonical division of labor holds, but the results are sufficient for locating windows of extreme leading-strandedness (green bars in **d**). These windows in turn yield estimates of the ribonucleotide incorporation rates for each polymerase (s_k in Eq. 3). (**e**) The polymerase-independent local noise factor, as calculated *pol3-L612G rnh201*Δ. (**f**-**g**) Estimated fraction of synthesis by each replicase on the top (**f**) and bottom (**g**) strands. (**h**) The deviation from expected Pol δ and α fraction of synthesis (DDAF), i.e. the fraction of synthesis not due to Pol ε. (**i**) A comparison of fractional top nascent leading strandedness ($F_{i\ lead,top}$), and fractional top strand synthesis by Pol ε ($f_{i,\varepsilon}$). (**j**) When these measures are plotted against one another, with $F_{i\ lead,top}$ as the independent variable, most points near the extremes lie on the diagonal of equivalence (black line; accounts for 1.14x scale difference; see Methods), but intermediate points are mostly below the diagonal. These intermediate points are found in termination zones. This suggests that Pol ε does less leading strand synthesis in termination zones than predicted by the canonical division of labor.

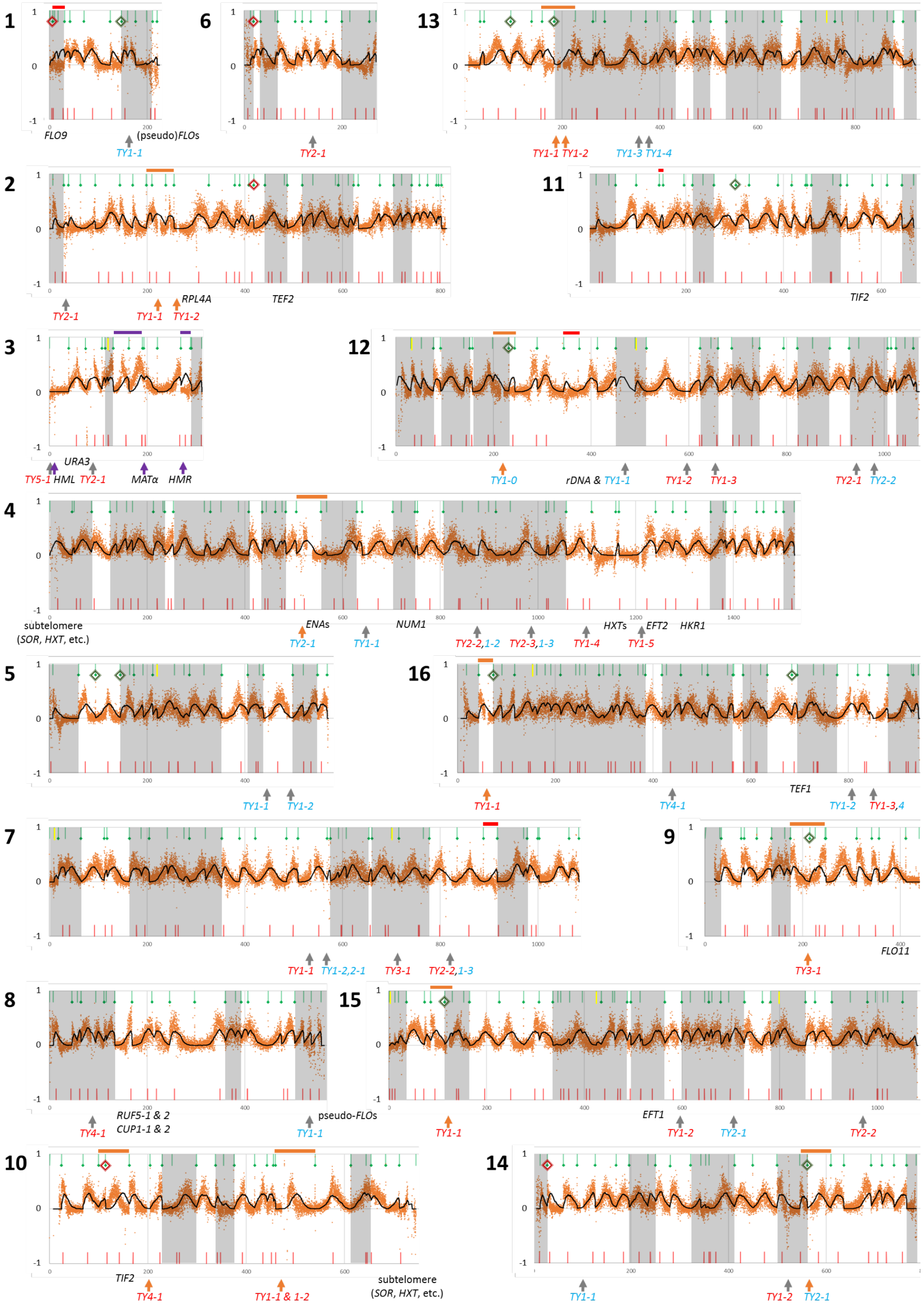
1
FLO9
(pseudo)FLOs
TY1-1
6
TY2-1
13
TY1-1 TY1-2
TY1-3 TY1-4
2
TY2-1
TY1-1 TY1-2
RPL4A
TEF2
11
TIF2
3
URA3
TY5-1 HML TY2-1
MATα
HMR
12
TY1-0
rDNA & TY1-1
TY1-2
TY1-3
TY2-1 TY2-2
4
subtelomere
(SOR, HXT, etc.)
ENAs
TY2-1
TY1-1
NUM1
TY2-2,1-2
TY2-3,1-3
TY1-4
HXTs
EFT2
HKR1
TY1-5
5
TY1-1 TY1-2
16
TY1-1
TY4-1
TEF1
TY1-2 TY1-3,4
7
TY1-1 TY1-2,2-1
TY3-1
TY2-2,1-3
9
FLO11
TY3-1
8
TY4-1
RUF5-1 & 2
CUP1-1 & 2
pseudo-FLOs
TY1-1
15
TY1-1
EFT1
TY1-2
TY2-1
TY2-2
10
TIF2
TY4-1
TY1-1 & 1-2
subtelomere
(SOR, HXT, etc.)
14
TY1-1
TY1-2 TY2-1

Supplementary Figure 3.

DDAF profile and simulated fork collision frequency across the *S. cerevisiae* genome. As per Fig. 1d but for all sixteen budding yeast chromosomes (indicated by large, bold numerals). DDAF (orange points), simulated fork collision density (black curve), origin positions (green bars with green diamonds for firing times established in advance, without for firing times were inferred herein), and predicted collision positions (red bars; given firing times and optimal global fork rates and assuming 100% origin efficiency) are indicated. Genome features with poor unique mapping of sequencing reads, resulting in low coverage, are indicated by name, as is *HMR*. Dark red/green diamonds indicate origins that fire later/earlier than expected, as indicated by bidirectional deviations between observed and predicted curves. Assuming a correlation between DDAFs and fork collision frequency, the orange points are also a comprehensive map of DNA replication termination zones.

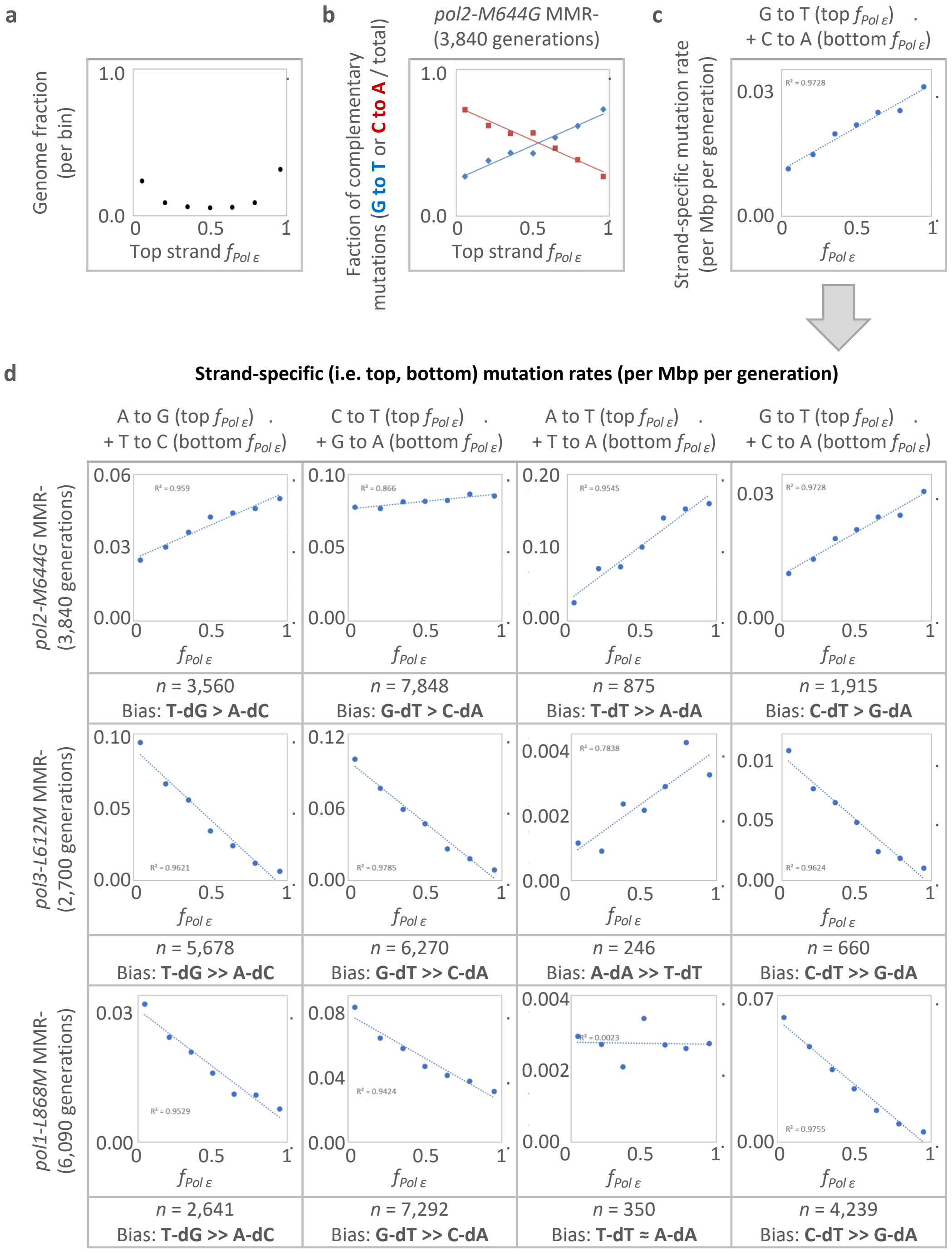

Supplementary Figure 4.

Characteristic mutation rates are linearly correlated with the DNA Polymerase ε synthesis fraction. For all graphs, the independent variable is the top strand Pol ε synthesis fraction ($f_{Pol\,\varepsilon}$). Mutations are from previous mutation accumulation experiments[5,6]. **a**, The genome fraction per

bin. **b**, **c**, Example from *pol2-M644G* MMR- budding yeast mutation accumulations. **b**, Fraction of complementary mutations and linear fits thereto: G to T (blue) and C to A (red). **c**, The strand-specific mutation rate: G to T measured versus the top strand $f_{Pol\ \varepsilon}$ and C to A measured versus the top strand $f_{Pol\ \varepsilon}$. **d**, As per panel **c** for three mismatch repair (MMR) deficient mutator replicase strains (genotypes listed on left) and for four complementary mutation pairs (listed along top).

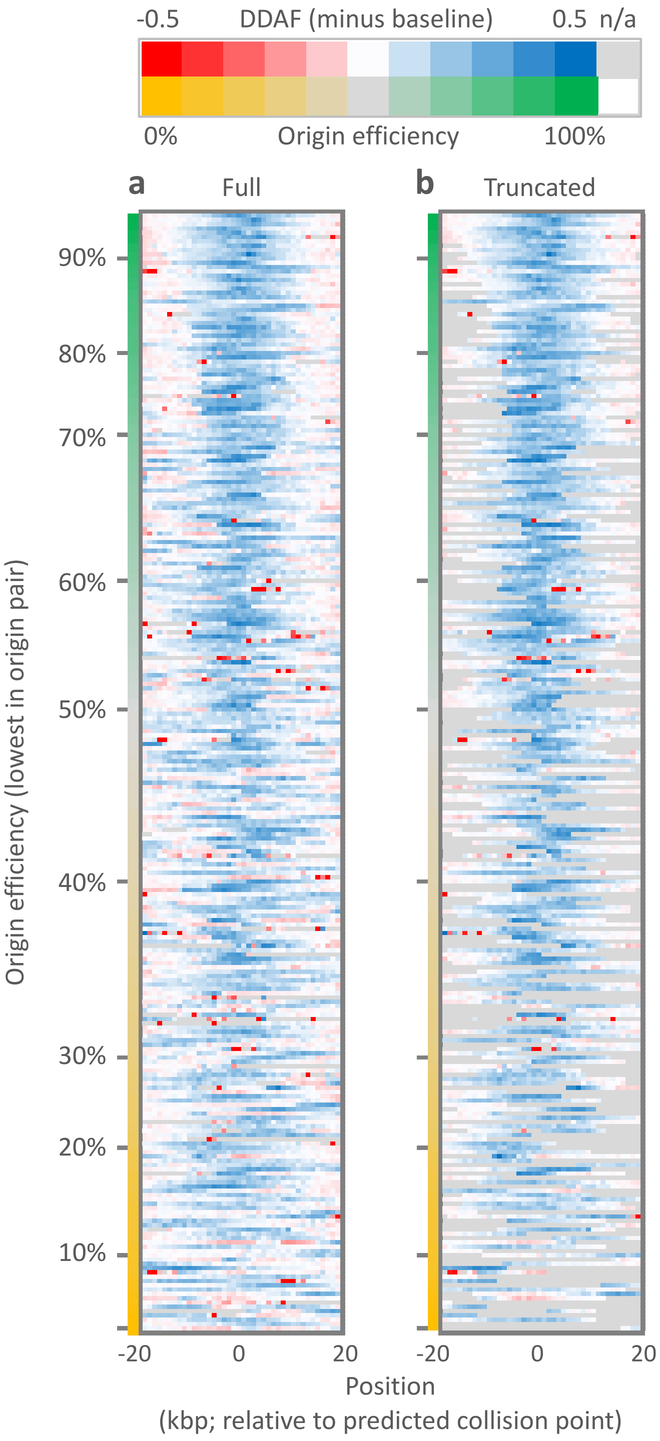

Supplementary Figure 5.

A comparison of DDAFs in predicted termination zones. DDAF heatmaps in 1000 bp bins for 20 kbp on either side of predicted collision points (red bars in Fig. 1d and Supplementary Fig. 3)

for forks proceeding from 259 well-separated adjacent origins (distance ≥ 20 kbp). Blue indicates a high DDAF (less Pol ε usage), red denotes the opposite. **a**, The full 40 kbp surrounding each predicted collision point. **b**, As per **a**, but truncated outside of the origin pair to prevent signals from adjacent inter-origin tracts from polluting averaged curves in Fig. 2f and 2g (orange curves).

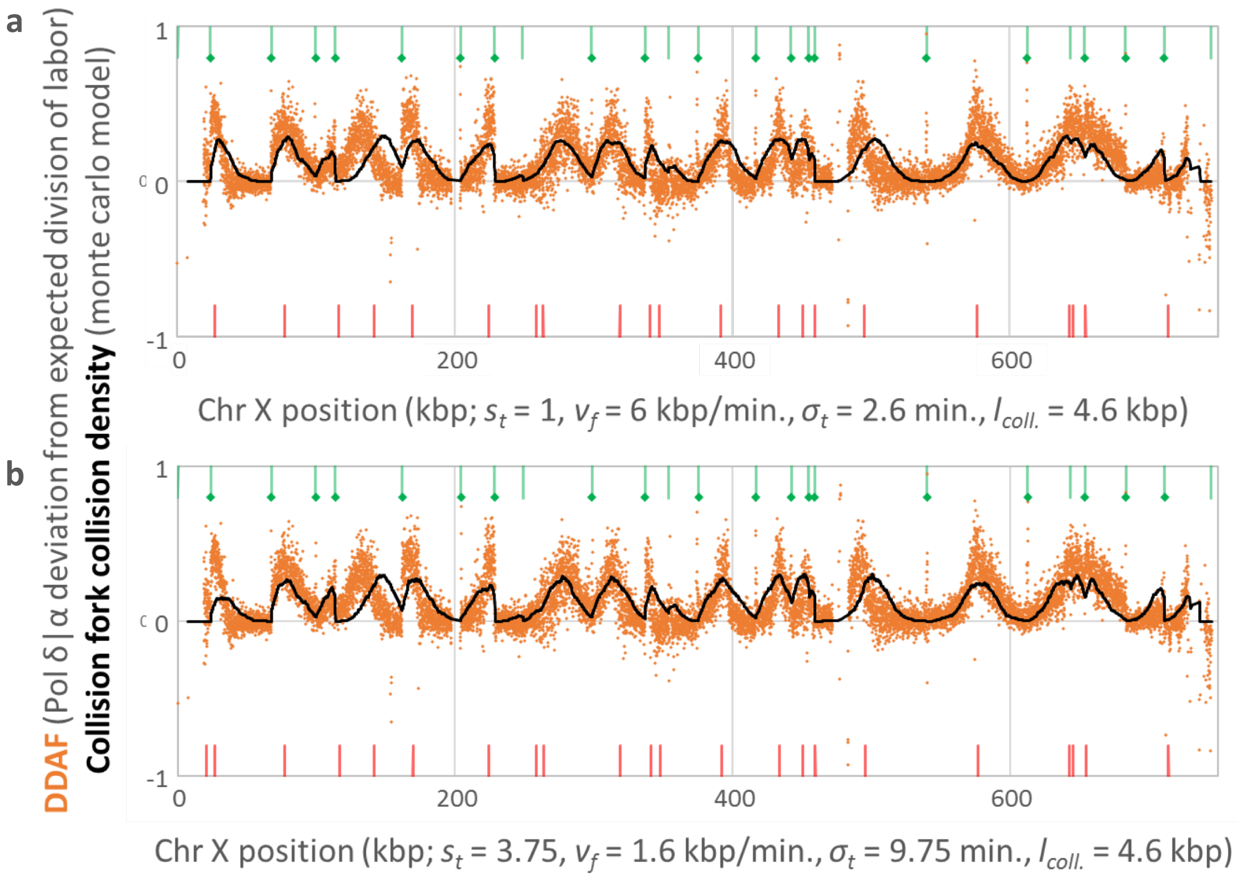

Supplementary Figure 6.

Simulated fork collision density given differing firing time scales. The DDAF (orange points) and fork collision density from Monte Carlo simulations (black curves; 1000 simulations each). Green bars represent origin positions, with green diamonds for firing times established in advance and without for firing times inferred herein. Red bars indicate predicted collision positions given firing times and optimal global fork rates and assuming 100% origin efficiency. Optimal simulation parameters are indicated below each panel: scale of firing times relative to previous estimates from α-factor arrest (s_t = 1 (**a**) or 3.75 (**b**) [5,7]); fork velocity (v_f); firing time deviation (σ_t); and collision tract length (4.6 kbp). Note that collision tract length is independent of firing time scaling. These Monte Carlo simulations are deliberately simple, with no tuning of listed origin positions or firing times, no locally variable fork speeds or firing time deviations,

and no allowances for DDAF peaks at origins or noise due to low coverage regions. Given the simplicity and measurement noise, the predicted termination profiles correlate remarkably well with DDAFs (R^2 = 0.578 with 1 kbp moving average of Chromosome IV DDAFs regardless of firing time scale; see Supplementary Fig. 7 and Methods for fitting parameters).

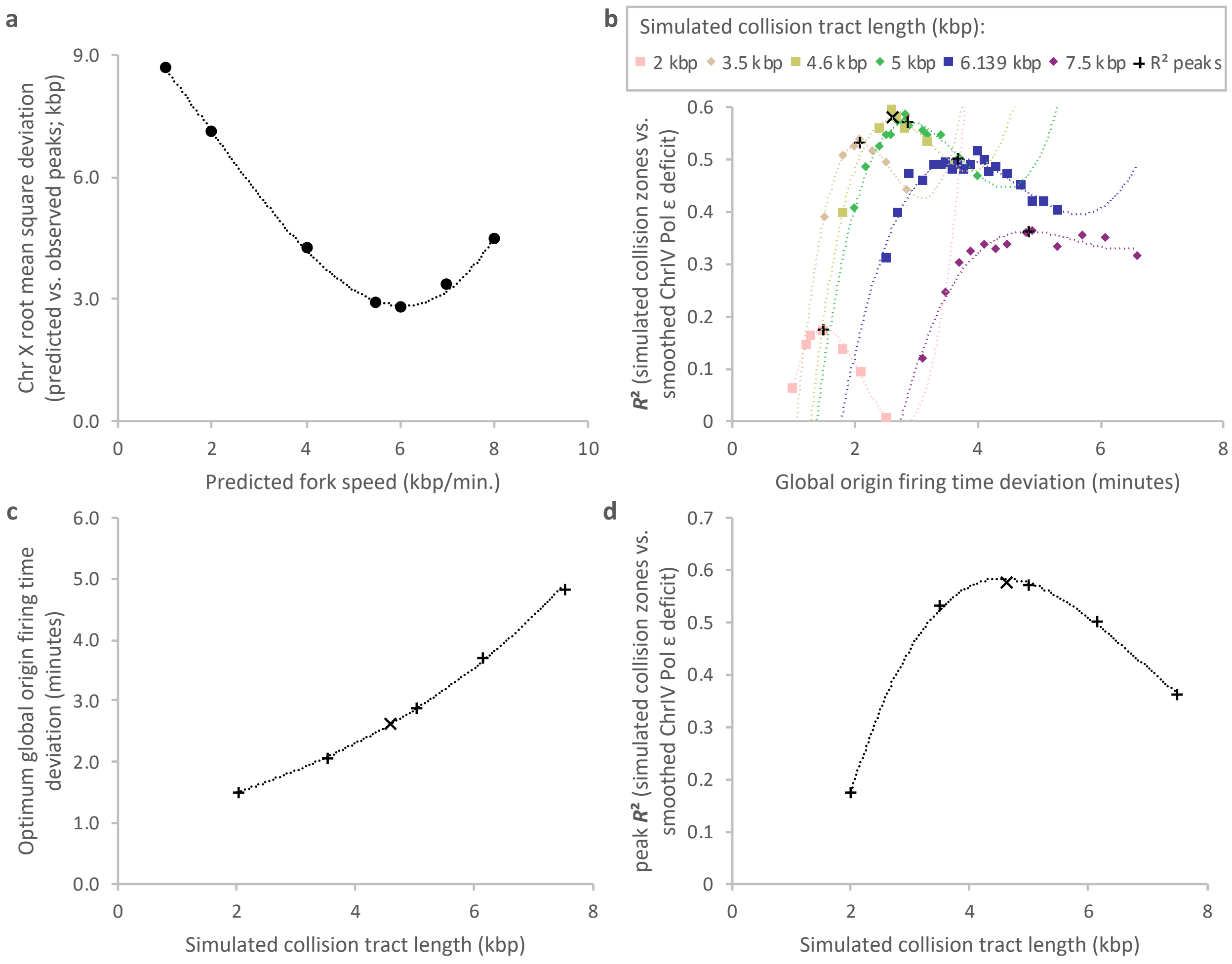

Supplementary Figure 7.

Choosing optimal fork collision simulation parameters to fit observed DDAFs. All curves are fits to third order polynomials except in panel **c**. Local extrema were calculated from same. The DDAF training set was from Chromosome IV (Fig. S2). **a,** The root mean square deviation between peak positions for DDAF and Monte Carlo simulated fork collision densities given differing global fork speeds (uses firing time list derived from α-factor arrest release[7] via ref.[5]. **b**, Coefficients of determination (R^2) between fork collision simulations and observed DDAFs (1 kbp moving average) given differing global origin firing time deviations and collision tract lengths. Local maxima are indicated by black crosses. **c**, The exponential relationship between

optimum (local maximum) global firing time deviation and collision tract length ($R^2 = 0.9996$).

d, Local maximum R^2 for fork collision simulations versus observed DDAFs given differing collision tract lengths. The local maximum is indicated by a black X and represents the fully optimized simulation.

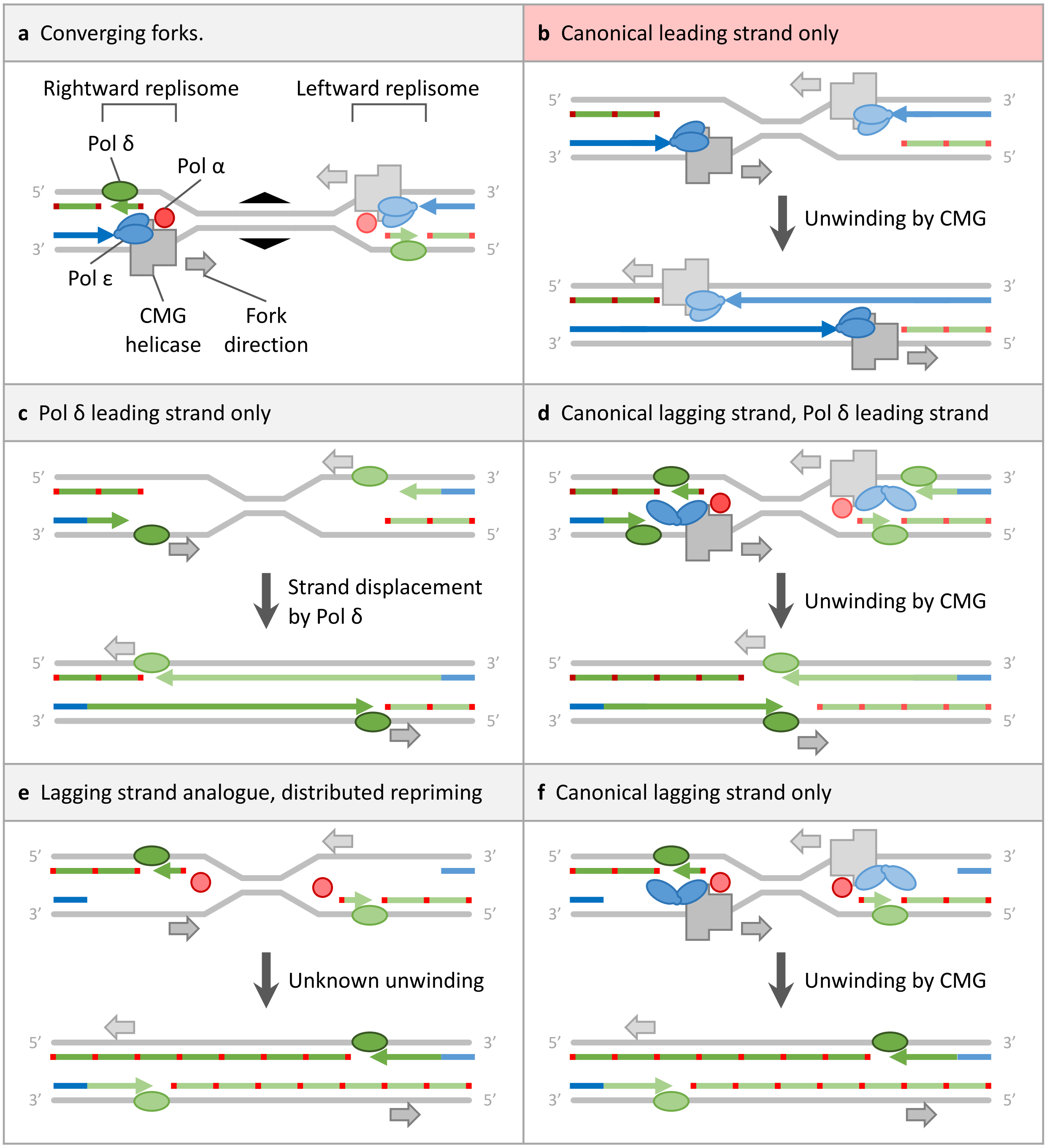

Supplementary Figure 8.

Models of polymerase usage in termination zones. Schematics of converging replication forks that are distant (**a**; canonical division of polymerase labor) or near to each other (**b-f**). Red, green and blue denote Polymerases α, δ and ε, respectively, or DNA tracts synthesized by same. DNA

strands (colored bars) and proteins are not shown to scale. Polymerases (ellipses) and CMG helicases (grey polygons) are exaggerated. Other replisome components are omitted for simplicity. **b**, Replisomes may proceed past one another without repriming, such that Pol ε synthesizes both strands (as previously suggested [8]). This scenario is not supported by our data. Depending on the involvement of Pol α we present four none mutually exclusive models **c**, Replisomes disassemble and Pol δ takes over from the 3' end of each nascent leading strand and continues until encountering the opposing lagging strand. **d**, Pol δ takes over the 3' terminus of nascent leading strand from Pol ε, while lagging strand synthesis continues as per usual. **e**, Replisomes disassemble and Pol δ continues, analogous to lagging strand Okazaki fragment synthesis but with distributed priming. **f**, Replisomes continue as normal but without Pol ε activity.

Scenario **c** is simple and analogous to both gap-filling re-synthesis during DNA repair [9,10] and extended Okazaki fragment synthesis. Presumably the last patch of synthesis in all scenarios would resemble Scenario **c**. Scenario **c** and Scenario **e** would require extensive Pol δ synthesis in cooperation with a helicase to separate parental strands. It has been suggested that the CMG complex remains associated with DNA until after the gap is filled and sealed [8]. Scenario **d** is analogous to replication restart after a fork barrier [11] and could proceed unidirectionally or bidirectionally. Scenario **f** is drawn as if repriming continues as normal, but it would more likely devolve into Scenarios **c** or **e** after parental strand unwinding and replisome divergence. Inactive Pol ε might remain bound to the helicase in Scenarios **d** and **f**. These scenarios are not mutually exclusive and may be distinguishable by the degree of Pol α usage. However, in the current RHII-HydEn-seq data, the Pol α contribution still shows noise levels compatible with even

extreme Scenarios **c** and **e**. Given that Scenarios **e** and **f** require extensive primase activity, which likely requires a functional replisome [12], we currently favor Scenarios **c** and **d**. Both are bolstered by evidence that Pol δ functions efficiently in the absence of a fully functional replisome under replication stress or DNA repair (ref. [11] and reviewed in ref. [10]). For reasons discussed about, Scenario **d** is preferred by the authors, but the others cannot yet be excluded.

Supplementary Table 1.

Oligonucleotides used for HydEn-seq library preparation. ARC140 containing a 5'-amino group is the 1st ligation adaptor. ARC76/77 duplex is the 2nd adaptor. ARC49 and indexing primer are used for library amplification. * indicates a phosphorothioate bond. Highlighted 6 nucleotide in the indexing primer is the index sequence.

ARC140	/5AmMC6/ACACTCTTTCCCTACACGACGCTCTTCCGATCT
ARC76	GTGACTGGAGTTCAGACGTGTGCTCTTCCGATCTNNNN*N*N
ARC77	AGATCGGAAGAGCACACGTCTGAACTCCAGTC*A*C
ARC49	AATGATACGGCGACCACCGAGATCTACACTCTTTCCCTACACGACGCTCTTCCGATCT
Indexing	CAAGCAGAAGACGGCATACGAGAT**NNNNNN**GTGACTGGAGTTCAGACGTGTGCTCTTCCGATCT

Supplementary References

1 Clausen, A. R. *et al.* Tracking replication enzymology in vivo by genome-wide mapping of ribonucleotide incorporation. *Nature structural & molecular biology* **22**, 185-191, doi:10.1038/nsmb.2957 (2015).

2 Itaya, M. Isolation and characterization of a second RNase H (RNase HII) of Escherichia coli K-12 encoded by the rnhB gene. *Proceedings of the National Academy of Sciences of the United States of America* **87**, 8587-8591 (1990).

3 Jinks-Robertson, S. & Klein, H. L. Ribonucleotides in DNA: hidden in plain sight. *Nature structural & molecular biology* **22**, 176-178, doi:10.1038/nsmb.2981 (2015).

4 Williams, J. S. *et al.* Evidence that processing of ribonucleotides in DNA by topoisomerase 1 is leading-strand specific. *Nature structural & molecular biology* **22**, 291-297, doi:10.1038/nsmb.2989 (2015).

5 Lujan, S. A. *et al.* Heterogeneous polymerase fidelity and mismatch repair bias genome variation and composition. *Genome research* **24**, 1751-1764, doi:10.1101/gr.178335.114 (2014).

6 Burkholder, A. B. *et al.* Muver, a computational framework for accurately calling accumulated mutations. *BMC Genomics* **19**, 345, doi:10.1186/s12864-018-4753-3 (2018).

7 Yabuki, N., Terashima, H. & Kitada, K. Mapping of early firing origins on a replication profile of budding yeast. *Genes Cells* **7**, 781-789 (2002).

8 Dewar, J. M. & Walter, J. C. Mechanisms of DNA replication termination. *Nat Rev Mol Cell Biol* **18**, 507-516, doi:10.1038/nrm.2017.42 (2017).

9 Prindle, M. J. & Loeb, L. A. DNA polymerase delta in DNA replication and genome maintenance. *Environ Mol Mutagen* **53**, 666-682, doi:10.1002/em.21745 (2012).

10 McVey, M., Khodaverdian, V. Y., Meyer, D., Cerqueira, P. G. & Heyer, W. D. Eukaryotic DNA Polymerases in Homologous Recombination. *Annual review of genetics* **50**, 393-421, doi:10.1146/annurev-genet-120215-035243 (2016).

11 Miyabe, I. *et al.* Polymerase delta replicates both strands after homologous recombination-dependent fork restart. *Nature structural & molecular biology* **22**, 932-938, doi:10.1038/nsmb.3100 (2015).

12 Yeeles, J. T. P., Janska, A., Early, A. & Diffley, J. F. X. How the Eukaryotic Replisome Achieves Rapid and Efficient DNA Replication. *Molecular cell* **65**, 105-116, doi:10.1016/j.molcel.2016.11.017 (2017).
