# Supplementary material for: Roles for DNA polymerase δ in initiating and terminating leading strand DNA replication
Source: Nat Commun. 2019 Sep 5;10:3992. doi: 10.1038/s41467-019-11995-z (PMC6728351; doi:10.1038/s41467-019-11995-z)
Supplement: Supplementary file 3 — Description of Additional Supplementary Files [file 41467_2019_11995_MOESM3_ESM.pdf]

Description of Additional Supplementary Files

File Name: Supplementary Data 1
Description: List of replication origins identified or verified in this study.
